# Mesh in rectopexy: biological, synthetic, or hybrid?

**DOI:** 10.1007/s10151-025-03266-5

**Published:** 2025-12-23

**Authors:** Marije A. Boom, Esther C. J. Consten

**Affiliations:** 1 Department of Surgery, UMC Groningen, Groningen, The Netherlands; 2 Department of Surgery, Meander Medical Centre, Amersfoort, The Netherlands

## Introduction

Two decades after the introduction of laparoscopic ventral mesh rectopexy (VMR) by D’Hoore and colleagues [1], the procedure has become a widely adopted treatment for rectal prolapse. Yet, despite its success in reducing recurrence and improving bowel function, a central controversy remains unresolved: *which prosthesis should be used?* The choice between synthetic and biologic mesh continues to divide opinion, reflecting the delicate balance between long-term durability and the risk of mesh-related complications [2].

A recently published prospective pilot evaluating OviTex^®^ PGA, a biologic graft reinforced with a fully resorbable synthetic scaffold, demonstrated technical feasibility and safety in VMR, with no erosions observed but some early recurrences [3]. Alongside this, Balaphas et al. revisit the broader controversies in rectopexy, emphasizing the persistent lack of consensus and the need for robust comparative evidence. Together, these contributions highlight both the progress made and the evidence gaps that remain.

## The core of the controversy: material choice

Synthetic polypropylene mesh has been the mainstay of VMR, offering strength, availability, and proven durability. However, rare but severe complications, including mesh erosion, fistulation, and chronic pain, have raised concerns among clinicians and the public alike [4]. Biologic prostheses, by contrast, are thought to be safer owing to gradual remodeling into host tissue, with large cohort studies reporting extremely low erosion rates [5–7]. Yet, this apparent safety may come at the expense of durability: recurrence rates appear higher in some series.

Meta-analyses reinforce this nuanced picture. van der Schans et al. found no significant difference in recurrence between synthetic and biologic meshes, though absolute complication rates were very low for both [8]. Similarly, a recent systematic review of more than 6000 patients by Hess et al. reported mesh-related complications in only 1.4%, independent of material [9]. In essence, the debate is not driven by high complication rates but by rare, severe events and the trade-off between safety and durability.

## What we learn from the new studies

The prospective pilot study of OviTex^®^ PGA offers important early insight. The absence of erosions is reassuring, while questions regarding durability remain. These results echo earlier findings from biologic cohorts, where safety was consistently high but recurrence rates varied [5–7]. As with many previous feasibility studies of novel prostheses, these data are encouraging but limited by small numbers and short follow-up. Nevertheless, pilot studies are a necessary first step to justify and inform the design of larger comparative trials.

The accompanying controversies paper by Balaphas et al. situates these findings in a broader context: despite the passage of time and increasing technical refinement, consensus on mesh selection remains elusive. Their call for robust comparative trials and international registries is timely and well-founded.

## Beyond material: a broader perspective

The debate cannot be reduced to “biologic versus synthetic” alone. Technique, fixation, suture material, and patient selection are equally important. The recent international expert consensus panel concluded that both biologic and synthetic meshes may be safely used, but the quality of evidence remains low and long-term follow-up is critical [10]. Importantly, suture choice may influence erosion risk as much as the prosthesis itself.

Variation in practice further complicates interpretation. A multicenter study from Belgium revealed striking heterogeneity in indications, fixation methods, and perioperative care across 14 hospitals [11]. This heterogeneity suggests that the lack of consensus is not solely about prostheses but reflects broader differences in surgical culture and clinical decision-making.

Cost considerations further complicate the debate. Polypropylene mesh is inexpensive and widely available, whereas biologic and hybrid grafts are substantially more costly. While cohort studies demonstrate excellent safety profiles for biologic meshes [5–7], it remains uncertain whether these advantages justify the financial burden, particularly in the absence of robust long-term comparative data [8, 9]. Recent consensus statements emphasize that cost and availability inevitably influence clinical decision-making, yet cost-effectiveness data are lacking [10]. Moreover, the broader societal and political scrutiny of mesh use has amplified the need to justify more expensive alternatives within constrained healthcare systems. Regulatory restrictions on synthetic implants in some countries, and the complex pathways for introducing biologic prostheses, further illustrate how policy and public perception intersect with scientific evidence [12]. In health systems under increasing economic pressure, cost-effectiveness will be an unavoidable part of the discussion.

Functional outcomes must also remain central. Several studies demonstrate that both biologic and synthetic meshes can yield substantial improvements in constipation, continence, and quality of life [6, 13]. Future research should therefore prioritize patient-reported outcomes alongside anatomical endpoints.

## The road ahead: reinforced biologic materials and trials

The introduction of reinforced biologic meshes, or hybrid constructs, such as OviTex^®^ 1S Permanent may represent a pragmatic middle ground, combining the safety profile of biologic grafts with the durability of limited synthetic reinforcement. This step follows naturally after the feasibility demonstrated with fully resorbable biologic grafts, which highlighted safety but also the need for improved durability. The key question now is whether reinforced constructs can deliver more sustainable long-term outcomes.

To address this, the ongoing ProTex trial (NCT06430931) is evaluating OviTex^®^ 1S Permanent against polypropylene mesh in robotic VMR and sacrocolpopexy. This multicenter, phase II–III, partially randomized patient-preference trial is powered for functional outcomes and designed to reflect real-world decision-making. Its results will provide much-needed comparative data and may guide the next phase of consensus-building.

## Conclusions

The debate over mesh in rectopexy remains unresolved. Synthetic mesh is durable but carries rare, serious complications; biologic grafts appear safer but may be less durable; and hybrid materials are promising but await long-term evidence. Recent meta-analyses confirm that complication rates are low across the board, yet the trade-offs remain clinically meaningful.

Consensus will not emerge from preference or tradition but from evidence. As reinforced biologic prostheses enter clinical evaluation, collaborative trials and registries will be crucial to determine whether they deliver on their promise. Until then, the choice of mesh in rectopexy remains a matter of careful, individualized judgment.

## Data Availability

No datasets were generated or analyzed during the current study.
